# Supplementary material for: Exploring women’s experience of healthcare use during pregnancy and childbirth to understand factors contributing to perinatal deaths in Pakistan: A qualitative study
Source: PLoS One. 2020 May 7;15(5):e0232823. doi: 10.1371/journal.pone.0232823 (PMC7205296; doi:10.1371/journal.pone.0232823)
Supplement: S1 File — (DOCX) [file pone.0232823.s001.docx]

| Participant ID: |
| --- |
| Date: _____/_____/_________ |
| Start time: _____:____am/pm  Finish time:_____:____am/pm |

**In-depth Interview Guide for women**

*(Stillbirth)*

**Section A: Introduction and Opening statement:**

Salutation

Information about the project.

**Section B: General & demographic information**

- Participant information

**Section C: Experience about stillbirth**

**Narrative account of the stillbirth and health services received during the pregnancy**

*Information about place of birth*

*Travel to health providers*

*Communities grief*

**Section D: Support during pregnancy and delivery**

- Support by families
- Support by other members of the community
