# Supplementary material for: Exploring women’s experience of healthcare use during pregnancy and childbirth to understand factors contributing to perinatal deaths in Pakistan: A qualitative study
Source: PLoS One. 2020 May 7;15(5):e0232823. doi: 10.1371/journal.pone.0232823 (PMC7205296; doi:10.1371/journal.pone.0232823)
Supplement: Attachment — Submitted filename: Response to Reviewers.docx [file pone.0232823.s002.docx]

18 December 2019

***Subject: Response to reviewers: PONE-D-19-26928_R1***

Dear Editor

We appreciate the comments from the reviewers which have helped improve our article. The updated

article has track changes showing the changes that we have made considering the reviewers’ comments.

In the updated version of the article, the line numbers, where reviewers had identified issues, are now

changed because of the text editing. Where applicable, we have now identified sections, and page and

line numbers for changes.

***Reviewer #1:***

1. I think there is a need to change the title of the study. Currently, it is little confusing.
Topic could be: Exploring women’s experience of healthcare use during pregnancy and childbirth to understand factors contributing to perinatal deaths

*Response: we have changed the title of the study as suggested.*

2. Talk a little bit more about your results in abstract section. You can reduce some text from background and methods if you like.

*Response: we have elaborated our findings in the abstract.*

3.I think you can further strengthen the rationale by explicitly stating the current gaps in the community-based maternal and child care services. Line no 100

*Response: we have added more information about current challenges to the maternal and child healthcare services (Introduction, Page 5, lines 120-127).*

4. Specify what factors, in particular, you would like to explore through this study? sociocultural? socioeconomic? religious? individual? biological? Line no 107

*Response: we have explained and elaborated the point about the objectives of this paper (Introduction, Page 6, lines 141-143).*

5. Line no 109. Within qualitative research, what design you employed to conduct this study? exploratory? descriptive? case study? etc.

*Response: the key word: “exploratory” is now included in the methodology section (Methods, Page 6, line 145).*

6. Under the settings heading, you have not mentioned why this Tando Muhammad Khan District has been selected?

*Response: we have added a line to explain the reason for selecting the district (Methods, Page 6, lines 150-151).*

7. Can you add the interview guides as appendix?

*We will include the interview guides, if the journal editors would like them, and that fits with in the journal guidelines.*

8. I couldn't see ethical considerations of this research in the methods section. Did you took consent with study participants?

*Response: ethical consideration was on title page, we have moved it to methods section in the updated version of the manuscript. Informed and written consent was taken and a statement is now included (Method, Page 10, lines 230-243).*

9. Regarding analysis, the FGDs and IDIs were analysed as one data set?

*Response: yes, there is this statement in the methods: “The IDIs and FGDs were analysed together”, (Methods: data analysis, page 9, line 205)*

10. I think it would be great if you could provide a table for study participants for FGDs and IDIs. Also, how many participants in one FGD?

*Response: We appreciate your suggestion for the table but a traditional tabular presentation of study participants in qualitative research may not be justified, as the numbers are small, and it doesn’t add anything to the manuscript as this information is clearly provided in the text.*

11. Also, the age and other demographic characteristics of participants that you have presented in results section could also be well presented in a table.

*Response: thank you for your suggestion. As explained in the response to the comment number 10, we prefer to keep demographic characteristics of participants as text. We initially presented these details as a table, but after consultation between senior researchers, the table was removed.*

12. I think you should rephrase your themes like unavailability of the healthcare services, poor access to care and poor quality of care. The themes should give a quick understanding about the study results.

*Response: we have changed the theme titles as advised.*

13. I think you can also make sub-themes within themes like high cost of care within poor access to care theme and so on.

*Response: we had sub-themes in the initial draft, however we merged them to give a bigger picture of the story that our research tells in response to the research questions. Subthemes did not add to the data analysis nor did they assist with interpretation and there was not enough data to make them substantive, therefore we would prefer to go only with main themes.*

14. Discussion section can be further strengthened by some good policy recommendation as in background section you stated that .. the purpose is to reorient policies and services

*Response: we have included a policy recommendation in the conclusion section (Discussion, Page 26, line 571-575).*

**Reviewer #2**:
Qualitative assessment of the healthcare use experience of women with a recent perinatal mortality in Sindh, Pakistan

1.First of all congratulations on a huge undertaking. To do this type of research in a developing country is extremely challenging. Obtaining Ethics committee approval, involving translation, and accessing 25 mothers in a rural community are wonderful accomplishments.
The description of your research process was clear and accurate. The participant’s comments were powerful and supported your conclusions and recommendations. The inclusion of health care providers was also important for validating what the mothers were saying. Excellent references!

*Response: the comments are extremely encouraging. Thank you.*

*2.A few areas might be better worded or clarified.
I am unclear as to who lady health workers are. I googled it and found out, but I wonder if a brief description of these women, with education and training details might be good.*

*Response: we have added details about LHWs in the methods (Methods, Page 7, lines 159-165).*

3.Line 104 is unclear to me. “we found that women with a high perinatal loss in Pakistan also have low empowerment” What is women with a high perinatal loss? Do you mean women who experience perinatal loss also experience lack of power? Maybe clarify.

*Response: we have clarified the sentence and elaborated it further.*

4.Line 235 Should the last word be “highly” rather than high?

*Response: we have edited the word as suggested (Results, Page 13, line 288).*

5.Line 401 is unclear to me. “Most women in Pakistan (82% using public care and 96% using private care) pay out of pocket for comprehensive emergency obstetric care service costs (16).” Might be clearer to say 82% of those using public care…

*Response: we have edited the sentence as: “About 82% women using public care and 96% using private care pay out of pocket for comprehensive emergency obstetric care service costs in Pakistan.” We hope that it is clearer now (Discussion, Page 21, line 454-456).*

6.Line 412 I am unsure of what you are saying here. “This study also showed that the effects on neonatal (and maternal) survival were much better in least developed countries” Could you clarify or explain further?

*Response: we have elaborated the text with more explanation* (Discussion, page 21 and 22, lines 466-468).

7.Thank you for this work. It is applicable to my work in a different developing country and I understand the great need. Your manuscript will help us continue to take steps that will impact the stillbirth rate worldwide!

*Response: thanks for the kind words, this is immensely motivating, and your words further strengthen my passion for working towards saving babies’ lives.*
