# Supplementary material for: Exploring women’s experience of healthcare use during pregnancy and childbirth to understand factors contributing to perinatal deaths in Pakistan: A qualitative study
Source: PLoS One. 2020 May 7;15(5):e0232823. doi: 10.1371/journal.pone.0232823 (PMC7205296; doi:10.1371/journal.pone.0232823)
Supplement: Attachment — Submitted filename: Response to Reviewers.docx [file pone.0232823.s003.docx]

**20 April 2020**

***Subject: Response to reviewers: PONE-D-19-26928R1***

Dear Editor

We appreciate the second round of comments, which have helped improve our article. The article is now updated following the comments in the second review. In the updated version of the article, the line numbers, where reviewers had identified issues, are now changed because of the text editing. Where applicable, we have now identified sections, and page and line numbers for changes.

Additional Editor Comments:

The topic of this manuscript is of utmost importance. However, manuscript's intelligibility is limited by the presence of grammar mistakes throughout the document. For instance, meaning of sentences in Line 126-128 and 132-136 is difficult to understand as they are currently written. I'd suggest having the manuscript revised by an English-proficient individual. Additional minor comments:

*Response: the article is thoroughly revised and checked for grammar mistakes.*

- Line 114: ‘other high perinatal burden countries’. I suppose the authors mean ‘other high perinatal mortality-burden countries’ as specified in line 119. Please correct.

*Response: we have corrected the specified errors and checked rest of the section for any additional errors (Introduction, Page 4, lines 78).*

- Line 123: ‘..management of server infections’. I suppose the authors mean severe. Please correct.

*Response: we have corrected the specified error (Introduction, Page 4, lines 87).*

- Line 193: please specify the occupation of the local Sindhi speaking interviewer at the time of the study and the type of training she received.

*Response: we have added details about the occupation, experience of the interviwer and the type of training she received for the data collection (Methodology, Pages 7-8, lines 156-162).*

***Reviewer #3:***

The authors performed a qualitative research on the causative factors of perinatal deaths in a rural area in Pakistan, interviewing women and families involved, as well as health care officers and lady health workers. I agree with the second reviewer on emphasizing the novelty and the effort of giving voice to women of this area, underlining social and economical problems of that society. However, as stated by Reviewer1, the manuscript needs to be reviewed in order to sound more scientific, although maintaining its qualitative nature. To do this, my suggestions are the followings (mainly in agreement with Reviewer 1):

• I suggest to add the interview guides as appendix: it would be useful for the scientific community in order to reproduce the same methodology for other qualitative studies on perinatal death.

*Response: As suggested, we have included the interview guides as appendix (Apendix-1).*

• I think it would be useful to provide a table for study participants for FGDs and IDIs, although the numbers are small.

*Response: we have included table-1 for the study participants.*

• Demographic data of participants are mandatory, presented in a table (age, BMI, parity, ...).
*Response: We have presented the demographic data of the participants in the results. This includes their mean age (SD), minimum and maximum age of the participants, and commented on the parity. Body Mass Index or parity or any other quantitative variables were not measured systemically because they were not part of our aims for the present study.*

Other minor changes:
• Introduction line 105: I would abbreviate in "Perinatal mortality is a challenge in developing countries..."

*Response: As a general rule, we have avoided using abbreviations since most terms are not repeated very frequently.*

• Introduction line 108: I would add "compared to 4 per 1000 births in high-income countries”.

*Response: We have added data about high income countries as advised* (Introduction: Page 3, Lines 71-73)

• Results line 256: I would use "delivered" rather than "birthed".

*Response: We have replaced "birthed" with "delivered" at places where the word was used.*

• Concerning grammar, pay attention to the use of articles: sometimes you can substitute "the women" with just "women". Please correct it in the manuscript.

*Response: We have removed unnecessary articles and made other grammar corrections.*

• Try to be more concise in the Discussion.

*Response: We have made changes in the discussion to make the section more concise. Any detail which was deemed extra is now removed.*
